# The Clinical Link between Type D Personality and Diabetes

**DOI:** 10.3389/fpsyt.2016.00113

**Published:** 2016-06-21

**Authors:** Chiara Conti, Danilo Carrozzino, Chiara Patierno, Ester Vitacolonna, Mario Fulcheri

**Affiliations:** ^1^Department of Psychological, Health, and Territorial Sciences, University “G. d’Annunzio” of Chieti-Pescara, Chieti, Italy; ^2^Psychiatric Research Unit, Mental Health Centre North Zealand, University of Copenhagen, Hillerød, Denmark; ^3^Department of Medicine and Aging, University “G. d’Annunzio” of Chieti-Pescara, Chieti, Italy

**Keywords:** diabetes, Type D personality, distressed personality, adherence, clinimetrics

## Abstract

**Introduction:**

Type D personality consists of a mixture of high levels of negative affectivity and social inhibition, resulting in a stable tendency to experience negative emotions, by inhibiting the expression of these emotions. We have reanalyzed the clinically relevant studies examining the role of this personality profile in diabetes, by providing a qualitative synthesis of the data. In this regard, the aim of this study is to provide a systematic review by evaluating the clinical link between Type D personality and diabetes.

**Method:**

When focusing on PRISMA guidelines, we have performed a comprehensive research of the literature on PubMed, Scopus, ScienceDirect, ISI Web of Science, PsycINFO, and Google Scholar by using search terms as “distressed personality” OR (i.e., Boolean operator) “Type D personality” combined with the Boolean “AND” operator with “diabetes.”

**Results:**

A total of seven research studies were identified and included in the review. Type D was found to be more prevalent in diabetes patients than controls. As regards the specific association with diabetes variables, Type D personality is a significant predictor of both poor medication adherence and unhealthy behaviors, by predicting negative mental health consequences also (i.e., depressed mood, anhedonia, and anxiety).

**Conclusion:**

Our review emphasized for the first time that Type D personality affects clinical factors in patients with diabetes by provoking adverse outcomes. The core implication of the study comprises the clinical relevance to detect, from a clinimetric point of view, Type D personality in diabetes in order to prevent potentially negative clinical outcomes.

## Introduction

When originally providing a theoretical definition of Type D personality or distressed personality, Denollet et al. (1) identified a combination of two stable personality traits of people who simultaneously experience high levels of negative affectivity in addition to social inhibition. That is, individuals having Type D personality show the specific tendency to experience negative emotions across time and situations by avoiding to express such emotions in social interactions due to fears of social rejections or any other type of disapproval from people (2). When taking the scientific evidence linking Type D personality with poor (i.e., negative) outcomes in several medical settings (e.g., cardiac populations and patients with diabetes) into consideration, the relevance of the current review paper comprises the study of the role (e.g., influence, mediating effect, correlation, and prevalence) of the distressed personality factor on the main diabetic clinical variables (i.e., glycemic control and self-management, medication adherence and treatment compliance, medical consequences and complications, diet, and healthy lifestyle and behaviors). When considering that Type D personality has been significantly associated with a more than threefold increased risk of negative medical outcomes, such as myocardial infarction, revascularization, and cardiac death, among subjects suffering from cardiovascular problems (3), since diabetes is entirely related to a clinically relevant risk of cardiovascular complications, Type D personality might also predict cardiovascular disease in diabetes due to the adoption of unhealthy behaviors (4). Therefore, by considering that generally people with Type D personality are reluctant to consult medical staff resulting in negative (i.e., adverse) clinical outcomes (5), whereas in diabetes the main therapeutic aim is represented by medical adherence, it is important to clinically detect the effects of this specific personality trait on diabetes prognosis. When mentioning the epidemiological data from Li et al. (6), reporting an estimated rates of 387 million people with diabetes in the world, it is highly relevant from a clinical point of view to clarify the effects of this personality construct for diabetes by taking into account the clinical relevance of a constant attention to diet, blood glucose monitoring, and medication consumption.

On this background, the aim of this manuscript is to provide new insights performing a systematic review by only reporting the clinically relevant studies analyzing the relationship (i.e., association, mediating role, and prediction) between the Type D personality, as conceptualized by Denollet et al. (1) and diabetes clinical factors.

## Materials and Methods

### Eligibility Criteria

Eligible articles included English-language papers published in peer-reviewed journals, reporting original data on the study of Type D personality in clinical sample of patients with diabetes having a documented medical diagnosis of diabetes type 1 or type 2, as certified by a clinical diabetes specialist, according to worldwide medically recognized recommendations and guidelines (7). As a direct consequence of the inclusion criteria reported above, we have excluded all studies analyzing the clinical relevance of Type D personality in other medical clinical settings, such as cardiovascular disease (i.e., patients with myocardial ischemia or infarction, coronary artery disease, heart failure, and cardiomyopathy), cancer (e.g., patients with colorectal cancer, tumor necrosis), neurological disease (e.g., patients with Parkinson, multiple sclerosis), and other different medical disorders (i.e., fibromyalgia, irritable bowel syndrome, patients with renal disease, or patients suffering from asthma). Furthermore, by considering our main aim to investigate the specific clinical consequences of Type D personality in patients with diabetes, we have excluded the research reports analyzing the relationship between Type D personality and metabolic syndrome in general population sample. The metabolic syndrome constitutes an extensively recognized significant risk factor for diabetes (8), provoking increased level of central fat deposition, glucose intolerance or insulin resistance, dyslipidemia, and hypertension as core clinical components linked to the potential consequent development of diabetes (9). However, when considering that not all patients with a metabolic syndrome have a diagnosis of diabetes, we have established, according to the aim of our systematic review, that our population target was specifically limited to clinical sample of patients reporting a medically referred diagnosis of diabetes without any type of restriction as regards the chronological age of the participants. Moreover, we have only included research articles reporting original data excluding by contrast all other different papers, such as reviews, meta-analyses, commentaries, letters to the editor, books or book chapters, conference abstracts/posters, and also manuscripts that were clearly irrelevant. Finally, studies were discarded whether full text was available or not.

### Information Sources and Searches

PubMed, Scopus, ScienceDirect, ISI Web of Science, PsycINFO, and Google Scholar databases were systematically searched from inception to April 2016. In addition, a manual search was performed by analyzing the bibliographical reference lists from all selected articles in order to identify other potentially relevant papers. The search terms, on which we have focused on, were “distressed personality” OR (i.e., Boolean operator) “Type D personality” combined with the Boolean “AND” operator with “diabetes.” When conducting a systematic review of the literature, the guidelines as included in the PRISMA statement (10) were followed. Titles and abstracts were screened by all authors in order to evaluate the validity of eligible studies according to our inclusion and exclusion criteria. Research reports evaluated as potentially relevant were retrieved, and all authors independently assessed each of the full articles in order to arrive at a consensus regarding eligibility of the selected papers. Any disagreements were resolved by final consensus.

### Analysis and Data Synthesis

By taking the heterogeneity of study design (i.e., cross-sectional or follow-up studies) into account, as well as the different administered measures of the several study protocols, it was not possible to combine the results into a meta-analysis. On this background, the methods described here fulfilled preferred reporting items for systematic reviews, because a meta-analysis was not deemed to be appropriate. Therefore, we have reported a qualitative synthesis of studies by performing a systematic review of the literature.

## Results

**Figure 1:**
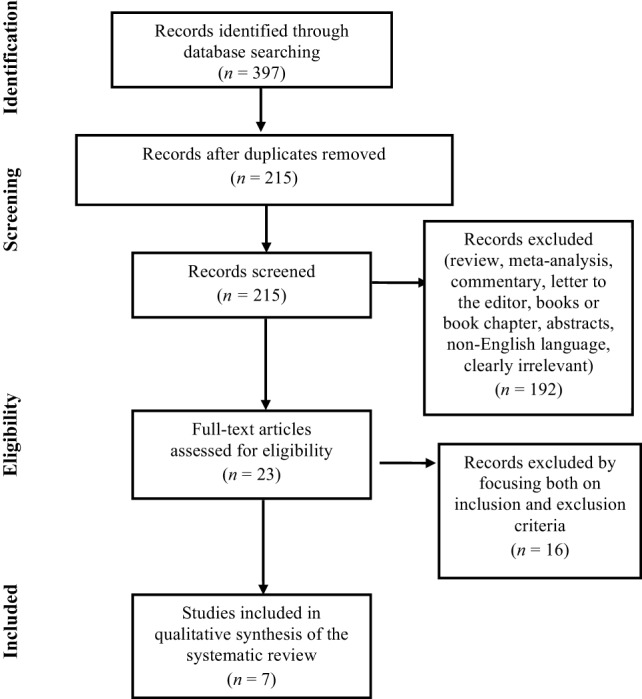
**PRISMA Flowchart of the systematic search**.

The search of PubMed, Scopus, ScienceDirect, Web of Science, PsycINFO, and Google Scholar databases initially provided a total of 397 citations, as reported in the PRISMA flow chart (Figure 1) showing the number of records identified, screened, included and excluded, and the reasons for the exclusions. After adjusting for duplicates and reviewing the titles and abstracts in order to exclude those that clearly did not meet the eligibility criteria, 215 remained. Of these, 192 studies were discarded because they did not meet the inclusion criteria. Of the 23 full text articles assessed for eligibility, 13 studies were excluded because they were not pertinent with the aim of our review study, and additional 3 studies were discarded because the full text of these papers was not available. Finally, a total of seven research studies were identified as clearly relevant and selected for inclusion in the systematic review, as briefly reported in the Table 1. No unpublished relevant studies were obtained.

**Table 1:** **Distribution of the seven relevant selected studies, including the reference, the research topics, the population/country, and the aims of the research**.

	Reference	Research topic	Population target/country	Aims
1	Nefs et al. (15)	Type D personality, suboptimal health behaviors and emotional distress in adults with diabetes	*N* = 3314 Type 1 or type 2 diabetes patients Netherlands	To evaluate the association between Type D personality and its dimensions with health behaviors, emotional distress, and biomedical risk factors
2	Nefs et al. (14)	Distressed personality and type 2 diabetes	*N* = 1553 Primary care patients with type 2 diabetes Netherlands	To investigate the main clinical correlates of Type D personality construct as assessed by DS14
3	Li et al. (6)	Association between distressed personality and medication adherence in patients with type 2 diabetes	*N* = 330 Patients with type 2 diabetes China	To explore whether Type D personality is a significant predictor of medication compliance
4	Milicevic et al. (11)	Personality traits and treatment compliance in type 2 diabetic patients	*N* = 164 91 with T2DM and 73 healthy subjects Bosnia and Herzegovina	To assess the clinical consequence of the Type D personality with respect to treatment compliance in diabetes
5	Nefs et al. (16)	Fear of hypoglycemia and type 1 diabetes	*N* = 288 Patients with type 1 diabetes Netherlands	To explore psychological factors (i.e., Type D personality), increasing risk of fear of hypoglycemia
6	van Dooren et al. (12)	Psychological and personality aspects in type 2 diabetes patients	*N* = 862 (609 = No type 2 diabetes; 253 = type 2 diabetes) Netherlands	To analyze psychological variables and personality traits affecting diabetes
7	Wiltink et al. (13)	Type D personality and diabetes	Total sample *N* = 5000 (*N* = 374 patients with diabetes) Germany	To study the association between Type D personality and diabetes

*T2DM, diabetes mellitus type 2*.

### Type D Personality and Treatment Compliance

By reporting a recent research study from Li et al. (6) aimed at investigating whether Type D personality was a predictor factor of poor medication adherence in a sample of 330 patients with a diagnosis of type 2 diabetes mellitus, the authors have found that patients with Type D personality scored significantly lower than participants without this personality trait on scores for medication adherence (*t* = 5.26, *p* < 0.001). Furthermore, by using multiple regression analyses, the authors were able to demonstrate that Type D personality predicted poor medication adherence rates (β = −1.48) before and after controlling for other covariates. In line with the previous research study, a report from Milicevic et al. (11), by focusing on a clinical sample of 91 type 2 diabetes mellitus participants (i.e., either inpatients or outpatients) matched with a control group of 73 healthy individuals, showed that Type D personality was significantly more prevalent (i.e., higher incidence) in the clinical sample of patients (i.e., 51.6%) than controls (i.e., 35.6%). In addition, Milicevic et al. (11) have found that diabetic patients with Type D personality were less compliant in terms of frequency of visits to the primary care physician (χ^2^ = 4.229, *p* = 0.040). As regards studies of prevalence analyzing the incidence of Type D personality in diabetes patients, another recent research report from van Dooren et al. (12), by performing an observational prospective population-based cohort study, including 253 patients with type 2 diabetes, have underlined that the levels of Type D personality were, from a statistical point of view, significantly higher in diabetic patients (*p* = 0.027) than controls, with a prevalence of 44 people with diabetes (i.e., 22.8%) having this personality trait. Furthermore, by testing the association of Type D personality with type 2 diabetes, van Dooren et al. (12) have found that the logistic regression analyses for Type D personality showed 95% higher odds for type 2 diabetes. Finally, when evaluating the limit deriving from a research report of Wiltink et al. (13) focused on a cross-sectional general population comprising 374 patients with diabetes, an inconsistent result was retrieved in contrast with the previous findings reported above, showing a non-significant association between Type D personality and diabetes.

### Type D Personality and Clinical Psychological Consequences

When focusing on 1553 primary care patients with type 2 diabetes in order to evaluate the main clinical correlates of Type D personality traits, a research study from Nefs et al. (14) highlighted that diabetic patients having Type D personality showed more loneliness and emotional distress, as well as higher levels of depressed mood, anhedonia, and anxiety (*p* < 0.001) than participants without Type D personality, indicating that this specific personality profile is also strongly associated with poor mental health.

### Type D Personality and Health Behaviors

When assessing whether Type D personality and its components (i.e., negative affectivity and social inhibition) are differently associated with health behaviors, emotional distress, and biomedical risk factors in adult patients with type 1 or type 2 diabetes (*N* = 3314), Nefs et al. (15) have found that participants with Type D personality reported significantly more barriers to taking medications than their non-Type D personality counterparts, by also showing a lower inspection frequency of the feet than respondents without Type D personality. Other interesting results derived from this recent research study (15) are the following: participants with Type D personality were less likely to meet the national recommendation for healthy exercise, as well as they show the tendency to consume unhealthy foods (i.e., non-lean meat, fried products, and sweets). Moreover, respondents having Type D personality were more prone to eat in response to emotional arousal. As regards the health care consultations, patients with Type D personality were less inclined to consult medical specialists during problems with diabetes management (15). When considering the association between diabetes and emotional distress, diabetes respondents with Type D personality showed more loneliness, symptoms of depression, and anxiety than the other groups (15). When analyzing the standard biomedical risk factors, diabetes adults with Type D personality reported a higher mean BMI and also higher levels of cholesterol than controls (15). Finally, when using multivariable analyses, Type D personality was significantly associated with suboptimal consultation behavior (OR = 2.03, 95% CI 1.69–2.44), with diabetes-specific social anxiety (OR = 2.85, 95% CI 2.35–3.45), with the presence of barriers to medication taking (OR = 2.86, 95% CI 2.36–3.47), and with suboptimal healthy eating (OR = 2.96, 95% CI 1.94–4.52). Furthermore, Nefs et al. (15) were able to demonstrate that the two Type D components (i.e., negative affectivity and social inhibition) also significantly increased the odds of the above-reported suboptimal behaviors (i.e., range of ORs 1.35–1.85 for social inhibition only and 1.36–2.38 for negative affectivity only), but the combination of negative affectivity and social inhibition resulting in the Type D personality consistently obtained the strongest independent association.

### Type D Personality and Fear of Hypoglycemia

When considering the scientific evidence that fear of hypoglycemia is associated with both anxiety and neuroticism, in order to test if Type D personality is also related to the fear of hypoglycemia in diabetes, Nefs et al. (16) have found that Type D personality appeared to be associated with the fear of hypoglycemia, but the association disappeared (β = 0.09, *p* = 0.195) when depressive symptoms were added to the regression model, indicating that the relationship between the two clinical factors could be accounted for by the depressive symptomatology.

## Discussion

To the very best of our knowledge, this is the first systematic review study aimed at investigating the published original research reports analyzing the clinically highly relevant relationship between Type D personality and diabetes in order to better understand the main clinical consequences deriving from this association. Although Type D personality may be considered as a relatively novel risk factor (17), its largely reported association with poor prognosis in diabetes (i.e., increased risks for adverse clinical outcomes) needs specific clinical attention (i.e., psychological assessment and treatment). Indeed, despite the growing body of studies reporting the harmful consequences of Type D personality in this patient group, the effect of Type D trait of personality have received relatively little research attention (18). On this basis, by evaluating the relevance regarding the evidence that patients with diabetes having Type D personality showed more difficulties to realize self-health management behaviors, it is clinically relevant to detect these personality factors early, namely the negative affectivity and social inhibition of the Type D personality in order to prevent potential risk consequences such as medical complications (i.e., neuropathy, nephropathy, retinopathy, glaucoma, hypertension, other macro-, and micro-cardiovascular problems) due to the poor adherence. To date, diabetes represents a prominent cause of vision loss, renal disturbances, and lower extremity amputations (19). Moreover, diabetes patients have to cope due to the disease progression with the long-term vascular complications (i.e., both macro- and microvascular problems). In this regard, when developing a prospective cohort study aimed to test if Type D personality affects the onset/progression of micro- and macrovascular complications in type 2 diabetes, Nefs et al. (20) have designed an original study protocol in order to provide an advancement of knowledge regarding the association between Type D personality and diabetes outcomes, by emphasizing the importance of examining both psychological and medical factors. On this background, bodies of studies (20) have confirmed the adverse effects of this personality construct not only on medical aspects but also on clinically important psychological factors (i.e., depression, anxiety, and anhedonia). When taking the very high prevalence (i.e., nearly 52%) of Type D personality among diabetic patients (11) into account, it is clearly important to prevent negative implications (e.g., avoidance of regular check-ups with family physician) associated to the presence of typical unhealthy behaviors, such as inhibition, self-absorption, and avoidant coping (21). Furthermore, by considering, as main limit of the examined studies, the very few research reports analyzing the effects of Type D personality on diabetes type 1 (15, 16), it is highly relevant to improve the study of the clinical implications of Type D personality on this sample in order to further confirm or not the results obtained with patients having diabetes type 2. By focusing on our preliminary results as qualitatively analyzed, Nefs et al. (15) have confirmed a high prevalence (i.e., 29%) of Type D personality both in patients with type 1 and type 2 diabetes, supporting a same effect of this personality profile on diabetes clinical variables. In this regard, future studies are recommended.

Finally, the major clinical implication of the current systematic review study comprises the relevance to screen for Type D personality in patients with diabetes in order to prevent potentially negative and adverse clinical outcomes by combining, within the scientific field of the clinical health psychology and medicine (22), the medical evaluation of the clinician with a specific clinimetric analysis (i.e., psychological assessment) of the psychological factors affecting diabetes (23). In this regard, a valid measure used to assess Type D personality in conjunction with the experienced judgment of the clinician is represented by the 14-item Type D personality scale (i.e., DS14) (2), whose items are rated on a 5-point Likert scale from 0 to 4 with a total score ranging from 0 to 28 for each subscale, namely negative affectivity (e.g., “I often feel unhappy”), and social inhibition (e.g., “I am a closed person”). Patients who score high on both subscales, as determined by a standardized cutoff score ≥10, are considered having Type D personality, as originally conceptualized by Denollet et al. (1). On this background, another potential future perspective comprises the objective to validate the clinical validity of the DS14 as screening measure in diabetes using items analysis, as established by Bech (24) with clinical psychometrics.

## Author Contributions

CC wrote the paper and provided substantial contributions to the conception and design of the review paper. DC wrote the manuscript and conducted the computer search by selecting clinical relevant research articles. CP wrote the paper by revising it critically for important intellectual content. EV gave clinical suggestions for the paper. MF gave final approval of the version to be submitted. Finally, all the authors have approved the final version of the manuscript and were accountable for the content of the work.

## Conflict of Interest Statement

The authors declare that the research was conducted in the absence of any commercial or financial relationship that could be construed as a potential conflict of interest.
